# Mounting Logan Crowns

**Published:** 1895-06

**Authors:** J. W. Heckler

**Affiliations:** Buffalo, N. Y


ARTICLE V.

### MOUNTING LOGAN CROWNS.

BY J. W. HECKLER, D. D. S., BUFFALO, N. Y,

For the central and lateral incisors it is preferable to mount the crown without a band, and my method is to prepare the root in the usual way, but to cut the labial side just a little beneath the gum margin, leaving the palatine portion as long as the judgment of the operator dictates. Select a crown that will not need any grinding on the cutting edge or the sides to make it fit, as it always impairs the beauty of a Logan crown to cut the surface. Also be extremely careful to select a crown with a neck similar to that of the root. This being done, grind the crown to as perfect an adjustment to the root as possible, taking care not to grind the platinum pin, as it would materially impair the strength of the crown. If the end of the root has been properly treated and filled, it is now ready for the final adjustment of the crown.

Take non-cohesive gold foil No. 4, fold it from 80-100 thickness, punch a hole in the center with a dam punch large enough to admit the pin, place the pad of foil on the crown, trim the ragged edges almost to the sides of crowy remove and place some thin cement in cup of crown, put pad back in place, tilt patient so the cement can be placed in root canals and all the air be expelled, otherwise the air acts as a cushion and keeps crown from going to place. All this being accomplished, place the crown in position, put a soft pine stick on the cutting edges of crown, and give a few sharp raps with the mallet to drive out all surplus cement and to condense the foil. Keep the crown firmly in place for a few minutes to prevent any movement, and it is advisable to have the patient remain a half-hour to allow the cement to thoroughly harden. Then burnish the gold foil hard around the joint and finish with disks and strips as you would a filling.

A crown set in this way does not show any cement whatever, and it makes a more perfect joint than is possible by any other method. It leaves no shoulder for the lodgement of food, is perfect in shape with the root, and hermetically seals the joint.—*Dental Digest.*

